# Neurocognition and Depressive Symptoms have Unique Pathways to Predicting Different Domains of Functioning in Major Depressive Disorder

**DOI:** 10.1177/07067437221133375

**Published:** 2022-11-21

**Authors:** Chelsea Wood-Ross, Tanya Tran, Melissa Milanovic, Ruzica Jokic, Roumen Milev, Christopher R. Bowie

**Affiliations:** 1 Department of Psychology, Queen’s University, Ontario, Canada; 2 Centre for Addictions and Mental Health, Ontario, Canada; 3 Centre for Neuroscience Studies, Queen’s University, Ontario, Canada; 4 Providence Care, Kingston, Ontario, Canada

**Keywords:** cognitive functioning, major depression, functional impairment, path analysis

## Abstract

**Background:**

Research has established the independent relationships between depressive symptoms to cognition and functioning in depression; however, little is known about the role of mediators in this relationship. We explored the role of neurocognitive abilities, depressive symptom severity, dysfunctional attitudes, and functional capacity in predicting two dimensions of daily functioning in individuals with major depressive disorder (MDD).

**Methods:**

One hundred and twenty-four participants (mean age  =  46.26, SD  =  12.27; 56% female) with a diagnosis of MDD were assessed on a standard neurocognitive battery, self-reported depressive symptoms, dysfunctional attitudes, and clinician-rated functional impairment. They completed a performance-based assessment of functional competence.

**Results:**

Confirmatory path analyses were used to model the independent and mediated effects of variables on two domains of functioning: social (relationships and social engagement) and productive (household and community activities). Cognition and depressive symptoms both predicted productive functioning, and dysfunctional attitudes mediated each of these relationships. Functional competence was a significant mediator in the relationship between neurocognition and productive functioning. Depressive symptoms and cognition were direct predictors of social functioning with no significant mediators.

**Conclusions:**

There are divergent pathways to different dimensions of daily functioning in MDD. Measurement implications include the consideration of multiple levels of predicting productive activities and more direct relationships with social outcomes. Treatments that directly target depressive symptoms and cognition might not generalize to improvements in everyday functioning if additional pathways to functioning are not addressed.

## Abrégé

**Contexte:**

La recherche a établi les relations indépendantes des symptômes dépressifs à la cognition et au fonctionnement dans la dépression; cependant, on en sait peu sur le rôle des médiateurs dans cette relation. Nous avons exploré le rôle des capacités neurocognitives, de la gravité des symptômes dépressifs, des attitudes dysfonctionnelles et de la capacité fonctionnelle dans la prédiction de deux dimensions du fonctionnement quotidien chez des personnes souffrant du trouble dépressif majeur (TDM).

**Méthodes:**

Cent vingt-quatre participants (âge moyen  =  46,26, ET  =  12,27; 56% femmes) ayant un diagnostic de TDM ont été évalués par une batterie de tests neurocognitifs standards, des symptômes dépressifs auto-déclarés, des attitudes dysfonctionnelles, et une incapacité fonctionnelle classée par un clinicien. Ils ont subi une évaluation basée sur le rendement de la compétence fonctionnelle.

**Résultats:**

Des analyses de confirmation de trajectoire ont servi à modeler les effets indépendants et médiés des variables sur deux domaines de fonctionnement social (relations, engagement social) et productif (ménage et activités communautaires). La cognition et les symptômes dépressifs prédisaient tous deux un fonctionnement productif et les attitudes dysfonctionnelles médiaient chacune de ces relations. La compétence fonctionnelle était un médiateur significatif dans la relation entre la neurocognition et le fonctionnement productif. Les symptômes dépressifs et la cognition étaient des prédicteurs directs du fonctionnement social sans médiateurs significatifs.

**Conclusions:**

Il existe des trajectoires divergentes vers différentes dimensions du fonctionnement quotidien dans la TDM. Les implications de mesure sont notamment la considération de niveaux multiples de prédiction d’activités productives et de relations plus directes avec les résultats sociaux. Les traitements qui ciblent directement les symptômes dépressifs et la cognition ne peuvent pas se généraliser en améliorations du fonctionnement quotidien si des trajectoires additionnelles vers le fonctionnement ne sont pas prises en compte.

The paths from cognitive deficits to impaired everyday functioning have been established in severe mental disorders such as schizophrenia and bipolar disorder.^1–3^ Well-replicated findings point to both direct effects of cognition on everyday functioning as well as the role of mediators such as functional capacity and mood symptoms in major depression. Despite remission from depressive symptoms, individuals with major depression continue to struggle with social functioning (e.g., maintaining friendships and getting along with other people) and productive activities (e.g., managing household tasks).^4–6^ Other disorders have shown unique functional outcomes with differential predictors and have explored cognition as both the content of thought (e.g., dysfunctional attitudes and beliefs about oneself) as well as cognitive processes (e.g., neurocognitive abilities in domains such as memory, attention, and executive functioning). Research demonstrates that a person's capacity to perform functional tasks might not directly map onto their performance. In this article, we sought to explore the predictors of social and productive functioning in major depressive disorder (MDD) to better understand the unique pathways to function.

Cognitive impairments (i.e., decreased concentration and indecisiveness) are part of the diagnostic criteria for MDD,^7^ and thus highlight the significance that cognitive dysfunction has on depressive psychopathology. However, studies to date examining the mechanisms of functional disability in unipolar depression have only examined bivariate relationships with cognition, leaving the role of potential mediators of the association between cognition and functioning in this population unexplained. Cognitive impairments are widely acknowledged as an important aspect of MDD.^8^ Meta-analyses of cognitive deficits in MDD reveal consistent and replicable impairment in the order of a small to medium effect size relative to healthy controls.^9–11^ Currently, depressed patients have been found to have statistically significant cognitive deficits of a moderate level of severity compared to healthy controls.^12^ Several findings support a mean impairment of 0.5-1.0 SD below the general population mean across attention, memory, processing speed, and executive functions,^10,13^ with approximately 25% to 50% of patients exhibiting deficits that are moderate-severe at 1 SD below the mean on at least one cognitive domain.^10,14^

Further, cognitive deficits are not an artifact of motivation or other depressive symptoms; they persist into remission, with up to one-half of remitted depressed patients continuing to show cognitive impairment.^15–19^ In a recent meta-analysis, it was found that cognitive deficits persist with a small to medium effect size following remission from a major depressive episode.^20^ These findings point to the pervasiveness and persistence of cognitive deficits in individuals with depression.

Cognitive functioning has been established as an important predictor of everyday functional skills and outcomes in populations with severe mental illness including schizophrenia,^21–23^ bipolar disorder,^3,24^ and depression.^25^ Recent work provides a foundational understanding of the direct and mediating effects of cognition on functioning in schizophrenia and bipolar disorder using statistical modelling.^1,2,26,27^ In both schizophrenia and bipolar disorder, the link between cognition and functioning is at least partially mediated by measures of functional competence—the ability to perform everyday living skills in a neutral environment, assessed with objective, performance-based role plays.^26,28,29^ In contrast, clinical symptoms are largely unassociated with cognition or functional competence, but do have a direct effect on real-world functioning. These findings of a mediated path from cognition to functional impairment in severe mental illnesses have spurred examinations of new treatment techniques that use the paths to sequentially address cognitive impairment and mediating variables to enhance real-world outcomes.^30^

Previous studies have explored the independent relationships of depressive symptoms to cognition and functioning in depression; however, little is known about the role of other mediators in this relationship. Dysfunctional attitudes are a core feature of depression, however, the mediating effect of these dysfunctional attitudes on the relationship between cognition and everyday functioning in depression has not yet been explored. In schizophrenia, higher dysfunctional attitudes have been correlated to lower functional capacity and worse everyday functioning,^31^ as well as greater cognitive impairment and worse social and vocational functioning.^32^ Additionally, dysfunctional attitudes have been shown to mediate the relationship between cognition and everyday functioning in schizophrenia.^33^

Limited research has explored functioning as a multidimensional concept in depression. In one study, both functional competence measures and self-reports of everyday functioning were used in a treatment-resistant depressed sample and found that deficits in sustained attention were associated with impaired social competence and recreational functioning, whereas executive functioning predicted impaired adaptive competence.^34^ The authors found that pathways to functional impairment are different depending on the domain of functioning explored. Taken together, there stands a need to examine potential mediators of how cognition relates to multiple aspects of functioning in MDD, including social (e.g., maintaining friendships and getting along with other people) and productive activities (e.g., managing household tasks).

In this article, we test the direct and mediating effects of cognition on everyday functional outcomes in MDD. Drawing from studies with other severe mental disorders, we hypothesized that functional competence would partially mediate the path from cognition to everyday productive activities, whereas dysfunctional attitudes would mediate the path from cognition to everyday social activities. We hypothesised that depressive symptom severity and dysfunctional attitudes would be directly related to everyday social activities and productive activities directly, without a meaningful relationship with functional competence.

## Method

### Participants

Participants in this study had a diagnosis of MDD and collapsed across cognitive remediation treatment trials. Participants were all outpatients referred by clinicians at community clinics and psychiatric hospitals. Inclusion criteria were a diagnosis of MDD, confirmed with the Mini International Neuropsychiatric Interview (M.I.N.I.)^35^ by Master's or PhD level students in clinical psychology, and age between 18 and 65. Participants with a diagnosis of MDD were included regardless of psychotic symptoms, but those with a diagnosis of a primary psychotic or neurocognitive disorder were excluded. Exclusion criteria also included an active substance use disorder, a diagnosis of schizophrenia or bipolar disorder, and neurological or physical conditions that would prevent valid assessment of measures or current engagement in treatment. Demographic information is provided in Table 1.

**Table 1.** Demographic Variables.

Category	
Age, *M* (SD)	46.26 (12.27)
Gender *n* (%)	
Male	51(41.13)
Female	70 (56.45)
Not reported	3 (2.42)
Population group *n* (%)	
White	96 (76.19)
Black	4 (3.17)
Southeast Asian	5 (3.97)
Chinese	6 (4.76)
Arab	2 (1.59)
Aboriginal	2 (1.59)
Not reported	11 (8.73)
Years of education, *M* (SD)	13.68 (2.59)
Cooccurring disorders *n* (%)	
Panic disorder	2 (3.45)
Obsessive-compulsive disorder	3 (5.17)
Posttraumatic stress disorder	2 (3.45)
Generalized anxiety disorder	14 (24.14)
Attention deficit/hyperactivity disorder	3 (5.17)
Past substance use disorder	19 (32.76)
Past alcohol use disorder	15 (25.86)
Medications *n* (%)	
SSRI	69 (54.76)
SNRI	24 (19.05)
Tetracyclics	13 (10.32)
Lithium	3 (2.38)
Antipsychotics	10 (7.94)
Benzodiazapines	7 (5.56)
Age at first treatment, *M* (SD)	24.02 (13.90)
Total number of hospitalizations, *M* (SD)	3.81 (5.41)
Total BDI score, *M* (SD)	18.66 (12.98)
WRAT *t*-score, *M* (SD)	53.18 (7.23)
DAS total, *M* (SD)	3.42 (1.17)
UPSA percent correct, *M* (SD)	83.83 (12.35)
NCS, *M* (SD)	41.10 (10.61)
WHO-DAS productive activities, *M* (SD)	2.63 (.83)
WHO-DAS social activities, *M* (SD)	2.21 (.78)

*Note.* SSRI  =  selective serotonin reuptake inhibitor; SNRI  =  serotonin and norepinephrine reuptake inhibitor; BDI  =  Beck depression inventory; WRAT  =  wide range achievement test; DAS  =  dysfunctional attitude scale; UPSA  =  University of California San Diego Performance-Based Skills Assessment; NCS  =  neurocognitive composite score.

### Procedure

All participants provided informed consent prior to completing the study. We received ethics approval from the Queen's University Health Science Ethics Board. Assessments were performed after participants provided written informed consent. A fixed assessment battery was delivered to all participants over two visits that were separated by a three-day period. Each visit lasted approximately 2 hours. Psychometrists were bachelor's or master's level staff who were trained to reliability by the senior author.

Neurocognition was assessed with a standard battery of tests developed for clinical trials of cognition, the MATRICS battery.^36^ This battery was designed following a consensus process to select tests that would be sensitive to measure a cognitive change in clinical trials. It includes tests measuring domains of attention/vigilance, processing speed, working memory, verbal learning, visual learning, reasoning, and problem-solving. T-scores (mean  =  50, SD  =  10) are calculated using a computer program that relies on normative data from a conormed group of healthy participants. Our primary variable was the Neurocognitive Composite Score (NCS), which is a global score with equal weight assigned to each cognitive domain, presented as a T-score. We did not administer the Mayer-Salovey-Caruso Emotional Intelligence Test, since previous work suggested that this social cognition measure did not load well with other domains on the MATRICS.^37^

Depression symptom severity was rated using the Beck Depression Inventory (BDI).^38^ This widely used 21-item self-report measure captures the broad symptoms of depression, with each item rated in severity from 0 to 3. Total scores range from 0 to 63, with higher scores indicating more severe depressive symptoms.

Defeatist beliefs were assessed using the Dysfunctional Attitudes Scale.^39^ Participants are asked to rate the degree to which they agree with maladaptive beliefs (e.g., “If I fail at my work, then I am a failure as a person”) on a 7-point scale, ranging from 1 to 7. Higher scores indicate more dysfunctional attitudes.

Functional competence was assessed with the University of California San Diego Performance-Based Skills Assessment (UPSA).^40^ This test uses role plays and structured questions to assess, in a neutral environment, the ability to perform everyday tasks such as household maintenance, shopping, planning recreation, and using money and transportation. Previous reports suggest that this measure mediates the relationship between cognition and functioning in schizophrenia and bipolar disorder.^1,28^ Percent of total correct was used as the dependent variable.

To measure overall functional impairment, we used the World Health Organization Disability Assessment Schedule II.^41^ This 36-item interview assesses six domains of functioning and provides detailed information regarding difficulties in these areas. The severity of difficulty is measured on a five-point scale, from 1 (*none*) to 5 (*extreme*). For the present analyses, we examined social and productive activity. Social activity was composed of the “getting along with people” domain and productive activity was composed of the “life activities” domain.

### Analyses

We used confirmatory path analysis to model the independent and mediated effects of variables on the two domains of everyday functioning (social activity and productive activity). Informed by previous studies in schizophrenia and mood disorders, we built a model with functional competence and dysfunctional attitudes as candidate mediators of cognition and depressive symptom severity. The goodness-of-fit of the models was tested and compared to several comparison models. The procedure relies less on the statistical significance of correlations among variables to determine their importance in predicting the outcome variable. To test the overall fit of the model, a comparison of models was made to an independent model, in which all variables are allowed to be correlated. The comparison models are made through the iterative elimination of nonsignificant paths from a saturated model (in which all variables are correlated). The best-fitting model was determined by examining several fit statistics: model chi-square, which compares the observed covariance matrix to the covariance matrix of the final model; the comparative fit index (CFI), which provides an estimate of the replicability of the model; and the root mean square of approximation (RMSEA), which is biased to favour a more parsimonious model. Model fit was considered good if the chi-square was not significant, CFI was at least .90, and the RMSEA was <.08. Analyses were conducted using SPSS, version 27 and its companion modelling software, AMOS, version 27.

## Results

One hundred and twenty-four participants completed all the baseline assessments. Demographic, clinical, performance, and functioning data are presented in Table 1. Bivariate Pearson correlations are presented in Table 2. Parametric comparisons of fit for the independent and final path models are shown in Table 3. Path coefficients are presented in terms of standardized regression coefficients originating from the final models.

**Table 2.** Bivariate Correlations.

	NCS	BDI Score	DAS Total	UPSA percent total	WHO-DAS social activities	WHO-DAS productive activities
NCS	1.00	—	—	—	—	—
BDI score	−.098	1.00	—	—	—	—
DAS total	−.288^a^	.250^a^	1.00	—	—	—
UPSA percent total	.532^a^	.016	−.250^a^	1.00	—	—
WHO-DAS social activities	−.284^a^	.580^a^	.263^a^	−.198^b^	1.00	—
WHO-DAS productive activities	−.339^a^	.382^a^	.398^a^	−.297^a^	.589^a^	1.00

*Note.* NCS  =  neurocomposite cognitive score; BDI  =  Beck depression inventory; DAS  =  dysfunctional attitude scale; UPSA  =  University of California San Diego Performance-Based Skills Assessment.

^a^ Correlation is significant at the 0.01 level (2-tailed).

^b^ Correlation is significant at the 0.05 level (2-tailed).

**Table 3.** Comparison of Models Developed to Determine Variables Predicting or Mediating Functioning Outcomes in Major Depressive Disorder.

Outcome domain and model	Chi-square	df	*p*	Comparative fit index	Root mean square error of approximation
Productive activities					
Independent	110.36	10	<.001	.00	.29
Iteration 1	4.60	3	.20	.98	.07
Iteration 2 (final)	7.58	4	.11	.96	.08
Social activities					
Independent	61.41	3	<.001	.00	.39
Iteration 1	2.01	2	.37	1.00	.005
Iteration 2 (final)	1.20	1	.27	1.00	.04

*Note.* The independent models posit that all variables are uncorrelated from each other. The final models develop from a saturated model (i.e., all variables are correlated) with nonsignificant paths eliminated until the best-fitting model is identified. The final model was a significant improvement from the independent model and had the best fit indexes of the iterations for every outcome domain.

For the productive activities and social activities outcome domains, the independent models were very poor fits to the data, with large and significant chi-square statistics and goodness-of-fit indices close to zero. For each outcome domain, a final model that fits that data was extracted from the saturated model. Both models of the outcomes had an excellent fit, with the final model superior to the independence model (Table 3).

**Figure 1.**
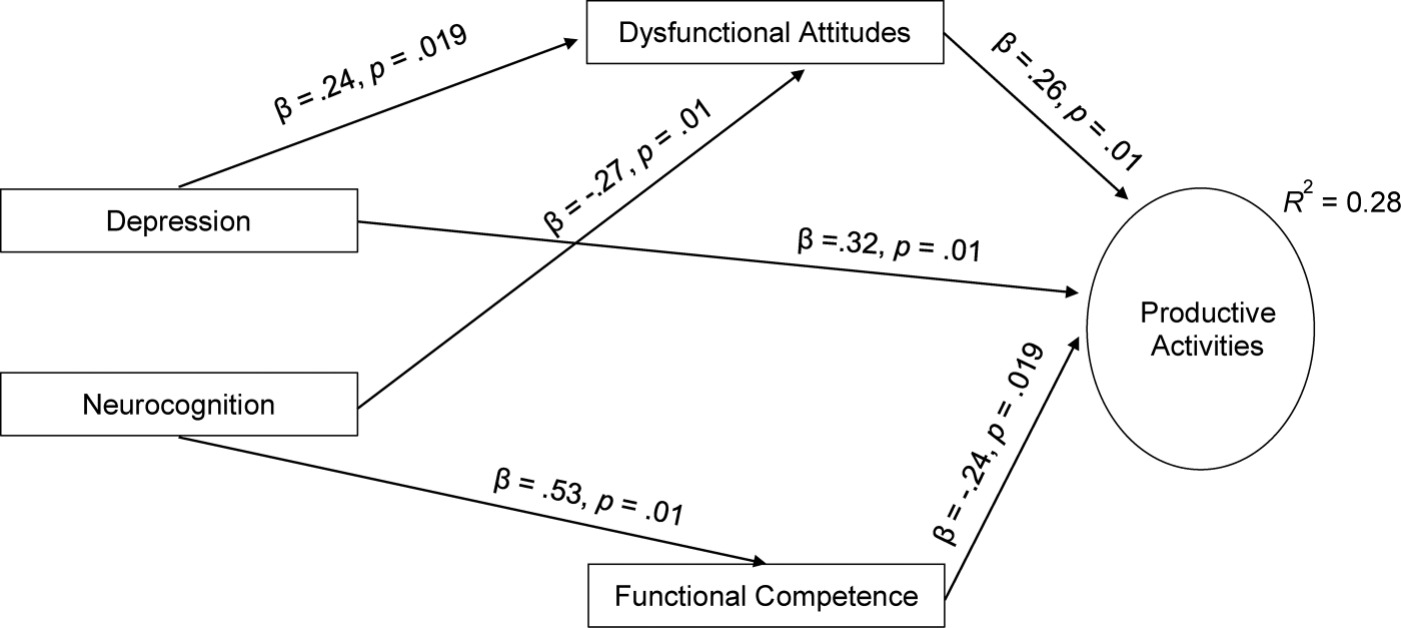
Final Path Model Predicting Productive Activity Impairment.

The best-fitting path model for productive activities is shown in Figure 1. This model has similar fit statistics to the first iteration and slightly smaller fit statistics; however, it was more parsimonious. In this model, productive activities were directly predicted by depressive symptoms. Depressive symptoms also predicted productive activities through a mediation effect of dysfunctional attitudes. Neurocognition had a relationship with productive activities that was mediated through both dysfunctional attitudes and functional competence.

**Figure 2.**
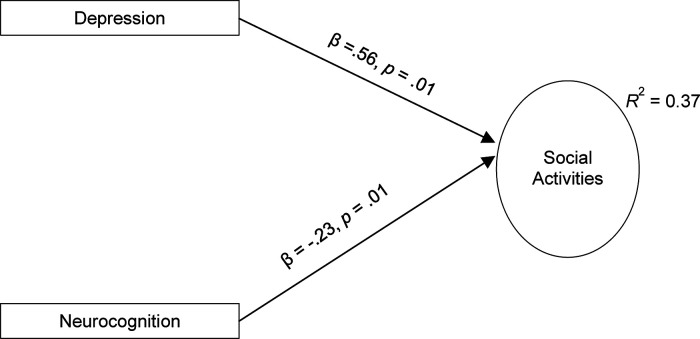
Final Path Model Predicting Social Activity Impairment.

The best-fitting path model for social activities is shown in Figure 2. In this model, social activities were directly predicted by depressive symptoms. Social activities were also directly predicted by neurocognition. There were no significant mediators.

## Discussion

In the present study, we explored the role of neurocognitive abilities, depressive symptom severity, dysfunctional attitudes, and functional capacity in predicting real-world functioning and social functioning in individuals with MDD. Similar to previous studies in depression^14,25,34^ we found a robust relationship between cognitive abilities and everyday functioning. Extending work from other severe mental illnesses,^1,2,26,27^ we also found that cognition was associated with both functional competence and everyday functioning.

There is mixed evidence^42^ for the primacy of overall depressive symptom severity versus cognitive impairment in predicting everyday functioning. Our results suggest that, like other studies, the relationship between cognitive ability and total depressive symptom severity was small. Our results are the first to show that with the modelling of direct and indirect relationships, there are divergent pathways that stem from root variables of cognitive impairment and depressive symptom severity to difficulties with functioning. Further, the pathways appear to be contingent on the specific type of functioning—social or productive. Neurocognition and depressive symptoms both predicted productive functioning, and dysfunctional attitudes mediated each of these relationships. Further, functional competence was a significant mediator in understanding the relationship between neurocognition and productive functioning. Thus, depressive symptoms have a robust direct relationship with productive functioning, whereas neurocognition is mediated by the functional skills that an individual has developed. In contrast to productive activities, depressive symptoms and neurocognition were found to be direct predictors of social functioning, while dysfunctional attitudes and functional competency were not.

Our study is among the first to demonstrate the paths between depressive symptoms, neurocognition, and social functioning in a sample of individuals with major depression. Few studies have demonstrated that social functioning is positively related to cognitive ability in MDD.^3,34,43^ A recent review highlighted that greater depression severity is associated with greater cognitive and psychosocial impairment, and thus may play a role in the relationship between cognition and functioning.^42^ However, we address a gap in the literature by testing this model and exploring possible mediators of these relationships in MDD. In a sample of individuals with schizophrenia, negative symptoms were demonstrated to interfere with interpersonal relationships, independent of neurocognitive ability and functional competence.^1^ Similar to our results, the authors also found that depressive symptoms limited interpersonal functioning.^1^ Our results add to the limited literature base in MDD and provide preliminary evidence that depressive symptom severity and neurocognition each independently predict social functioning, and clinical features of depression—specifically dysfunctional attitudes—do not mediate the paths between depressive symptoms and neurocognitive ability to social functioning in MDD.

Our study is one of the first to explore the relationships between cognition, functioning, and underlying beliefs about oneself in depression. Experimental psychopathology studies have explored reactions to performance on cognitive tasks in individuals with depression. Those with depression have been shown to have a significantly decreased performance to perceived failure on cognitive tasks.^44^ Similarly, other research has demonstrated negative perceptions of performance on cognitive tasks despite no performance differences to healthy comparison participants.^45^ Our results extend this previous work and demonstrate that dysfunctional attitudes, or an individual's negative underlying beliefs about themselves, mediate paths to productive functioning in total depression symptom severity and neurocognitive abilities. Further, we found preliminary support for depressive cognitions mediating the relationship between neurocognition and depressive symptoms to productive functioning in depression. These results suggest that individuals' negative underlying beliefs about themselves may be a particularly important barrier to productive functioning in depression.

Study results should be interpreted within the context of limitations. The ratings of functional capacity came from a psychometrically sound measure,^40^ however, some of the living skills that are assessed through this measure have lower priority in contemporary society (e.g., cheque writing and calling a phone operator). Additionally, there are possible mediators that were not included in the analysis, including social competence. Future research may aim to use an updated functional capacity assessment that includes an assessment of social capacity as well. All patients were seeking treatment to help improve their cognitive difficulties, and thus the role of cognition should be considered in this context as the results of this study may not be generalizable to a broader MDD sample. The sample had a relatively smaller range of cognitive impairment which may thereby limit the generalizability of the model of functioning.

In summary, the results of our study are among the first to demonstrate multivariate path models linking neurocognition, functional competence, and depressive symptoms with multiple domains of functioning in major depression. We found divergent paths to functioning in MDD; productive activities are multiply determined by both direct and mediated paths whereas social functioning was predicted by direct paths. Targeting depressive symptoms and neurocognitive abilities may improve outcomes for both social and productive domains of functioning. In contrast, targeting dysfunctional attitudes and functional skills in treatment may result in subsequent improvements in productive functioning. These results have implications for treatment planning in MDD and provide preliminary areas to target that may be obstacles to functional recovery. Current treatments, such as cognitive remediation, that target cognition and functional skills might be particularly important in achieving functional recovery in MDD. Specifically, the results from our study suggest that cognitive remediation programs that focus solely on restorative techniques with drill and practice may result in proximal improvements in cognition, but may have fewer distal implications for improvements in everyday functioning given that this pathway seems to be mediated by attitudes and functional capacity The results of this study also suggest that these existing behavioural treatments may need to additionally target depressive cognitions to result in the best functional outcomes.

### Data Access

Our consent forms at the time of the data collection did not include information about open data access and so raw data are not available. The authors will be able to provide summary statistics at request.
